# Factors influencing reproductive choices of HIV positive individuals attending primary health care facilities in a South African health district

**DOI:** 10.1186/s12889-017-4432-3

**Published:** 2017-06-02

**Authors:** Samuel Agbo, Laetitia C. Rispel

**Affiliations:** 1 Department of Family Medicine, Faculty of Health Sciences, University of the Witwatersrand, Johannesburg, South Africa; 2 School of Public Health, Faculty of Health Sciences, University of the Witwatersrand, Johannesburg, South Africa; 3 Centre for Health Policy & DST/NRF Research Chair, School of Public Health, Faculty of Health Sciences, University of the Witwatersrand, Johannesburg, South Africa; 4 P.O BOX 12089, Tramshed, Pretoria, 0126 South Africa

**Keywords:** Reproductive choices, HIV positive patients, Desire for children, HIV&AIDS, South Africa

## Abstract

**Background:**

There is global recognition of the reproductive health rights of people living with HIV (PLHIV). The aim of this research study was to explore the reproductive choices, and the factors influencing these choices, of HIV positive patients attending primary health care (PHC) facilities in the Ekurhuleni health district of the Gauteng Province of South Africa.

**Methods:**

During 2013, a cross-sectional survey was conducted in the Ekurhuleni health district. At each of three randomly selected community health centres, a random sample of HIV positive patients was selected. After informed consent was obtained, trained fieldworkers administered a structured questionnaire that elicited information on socio-demographics, reproductive choices and knowledge of reproductive options. Survey data were analysed using STATA® 13.

**Results:**

The majority of survey participants (*n* = 430) were female (70%) and unemployed (57%). The mean age of participants was 36.4 years (SD 8.6): 40.8 years (SD 8.7) for men and 34.5 years (SD7.8) for women.

Among survey participants, 46% expressed a desire for children (95% CI: 41.4–50.9). In the multiple logistic regression analysis, predictors of desire for children were age less than 49 years, marriage or living together, and no biological children. The odds of wanting children was 16.48 [95% CI: 5.94–45.74] times higher for PLHIV without children, compared with those with two or more children, while for those less than 25 years, the odds of wanting children was 0.78 [95% CI: 0.23–2.59] compared with those older than 50 years.

The PLHIV knowledge on the available reproductive options was limited, with the majority relying on the guidance of the health workers.

**Conclusion:**

Health care providers at PHC level should be educated to address the reproductive health needs of PLHIV. These aspects should be reflected in provincial and national health policies.

**Electronic supplementary material:**

The online version of this article (doi:10.1186/s12889-017-4432-3) contains supplementary material, which is available to authorized users.

## Background

The HIV & AIDS epidemic continues to rank among the top ten priority conditions that contribute to the global disease burden [1], with sub-Saharan Africa accounting for 66% of the global HIV burden [2]. Although there is new optimism of achieving an AIDS-free generation by 2030 [3], there is also recognition that a rapid scale-up of existing programmes is needed to achieve this ambitious goal [3, 4]. South Africa continues to be a priority country for intervention [4], with an estimated 6.4 million People Living with HIV (PLHIV) [5–9].

Sexual transmission of HIV infection continues to account for the majority of global HIV infections [10]. The improvement in HIV management especially the introduction of anti-retroviral therapy (ART) has resulted in improved quality of life and increased life expectancy of HIV positive persons [4]. In recent years, the sexual and reproductive health rights of PLHIV have been re-iterated, especially the responsibilities of governments to ensure that health care and legal systems support these rights [11]. Consequently, there has been the re-emergence of debates regarding reproductive choices and desires of HIV positive people [12–14]. The WHO has indicated that the unmet need for family planning remains high among HIV positive women, and has emphasised the strengthening of linkages between HIV and sexual and reproductive health programmes [4].

There is a significant body of literature on reproductive choices of and challenges faced by PLHIV [15–25]. Existing evidence suggests that a sizable proportion of PLHIV, regardless of geographical location, have an expressed desire for biological children [17, 21–27]. Factors that influence this desire included younger age, personal health status, having a regular partner, perception that the partner wanted children, knowledge of ART effectiveness, ethnicity, knowledge about contraception or the cultural issues [17, 24, 26, 27]. In sub-Saharan Africa, studies have reported the immense pressure on women to have children regardless of their HIV status [17, 19, 21, 23]. However, another study found that the situation for men was grave as well, with not having children often resulting in stigmatisation and loss of social status among peers [28].

Several studies have found that the attitudes of health care providers influence the reproductive choices of PLHIV [19, 29–35]. Many PLHIV want to discuss sexual and reproductive health needs with their health care providers, but reportedly feel uncomfortable about doing so [19, 29–35]. The study findings suggest that access to sexual and reproductive health services is critical, while advocacy groups have argued that weak health systems deny PLHIV their sexual and reproductive health and rights [11].

In South Africa, major health sector reforms towards universal health coverage (UHC) underscore the centrality of primary health care (PHC) [36, 37]. It is envisaged that a comprehensive range of health care services, including sexual and reproductive health services, would be delivered at PHC level [37]. In light of the global emphasis on addressing the sexual and reproductive health needs of PLHIV, and the importance of PHC to achieving UHC, the aim of this research study was to explore the reproductive choices, and the factors influencing these choices, of HIV positive patients attending primary health care (PHC) facilities in the Ekurhuleni health district of the Gauteng Province of South Africa.

## Methods

### Study setting

The study was conducted in the Ekurhuleni health district, one of the five health districts in the Gauteng province of South Africa [38, 39].

In 2013, the district had an estimated population of 3 million people and the PHC facilities head count was 5 million with utilisation rate of 2 million, of the public health sector facilities [40, 41]. The Ekurhuleni district health system (DHS) consists of a district hospital, seven community health centres (CHCs) and 87 PHC clinics. These PHC facilities (CHCs and clinics), are staffed primarily by professional nurses, who are supported by generalist medical officers and family physicians. Health care services are provided free of charge i.e. there is no out of pocket payment at these PHC facilities [40].

### Ethical considerations

The study was approved by the University of the Witwatersrand’s Human Research Ethics Committee and the provincial health authority. Standard ethical procedures were adhered to, including detailed participant information sheets, obtaining informed consent and ensuring confidentiality of information. A written consent document was signed by all participants who agreed to take part in the research. A distress protocol was developed, and any individuals who needed support and counselling because of emotional distress experienced during data collection were referred to the lay counsellor based at the health facility.

### Study population

The study population consisted of all the HIV positive patients attending public sector PHC facilities within the Ekurhuleni district. In 2013, 143,871 patients were on ART and cared for at these PHC facilities [40]. Each community health centre (CHC) had an average weekly head count of 500 HIV positive or 2000 patients per month, and an average female to male ratio of 70:30 [40, 41].

### Study design and sampling

During 2013, a cross-sectional study was conducted to explore the reproductive choices of HIV positive patients in Ekurhuleni district of Gauteng province, and the factors influencing these choices.

The sampling frame consisted of all HIV positive patients attending the seven CHCs in Ekurhuleni heath district. The CHCs were selected as they are well resourced, provide comprehensive PHC services, have dedicated HIV clinics and attend to a large number of the HIV positive patients.

The required sample size for the study was 369 patients, which was adjusted to 442 to take account of a possible refusal rate of 20%. The eligibility criteria for participation included known HIV-positive patient in the district, 18 to 49 years in the case of women, and 18–60 years in the case of men. These age groups are the bulk of PLHIV in their most reproductive period of life. All patients who were too sick to be interviewed or who declined participation were excluded from the study.

One CHC from each of the two sub-districts - east and south was sampled randomly while the only CHC from the north was selected. The study participants were selected from these sampled CHCs, using systematic sampling with a random start, until the required sample size was reached.

### Data collection

A pre-tested semi-structured questionnaire, developed in line with the study objectives, and translated into isiZulu and seSotho, the two major local languages, was used to collect data. The questionnaire obtained information on socio-demographic characteristics, HIV treatment, reproductive choices, and knowledge of reproductive options (Additional file 1). Following introduction by a nurse at the selected CHC, the two field workers proceeded with the recruitment processes. These field workers completed secondary school education, and were given both theoretical and practical research field work training.

At each CHC, the questionnaire was administered by these trained field workers (one female and the other male) after informed consent had been obtained. The field workers ensured that each person’s privacy was maintained.

### Data analysis

The data were captured in Microsoft excel and analysed using STATA® 13. Frequency tabulations were done to describe the socio-demographic characteristics of the respondents. In the inferential statistical analysis, the variables examined included the following: associations between socio-demographic characteristics such as: age, marital status, number of children, and level of education, employment and home language, and reproductive desires, reasons for reproductive desires, availability of reproductive services, knowledge of reproductive options, and disclosure of HIV status.

The Chi square test was used to determine the level of association of the factors influencing decision to have a child / children. Univariate logistic regression models were fitted to find factors, which were independently associated with desire to have children. Only factors associated with desire for children that were found to be statistically significant were considered in the model building exercise using a multiple logistic regression model. All statistical tests were carried out at 5% significance level.

## Results

### Socio-demographic characteristics of study participants

The majority of study participants (70%) were women. The mean age of all participants was 36 years (SD 8.6). The mean age of men was 40.8 years compared to 34.5 years for women, and this difference was statistically significant (*p*-value <0.05). Marital status also differed significantly by gender with women (49%) being more likely to be single than men (33%). The majority of the participants have either completed primary or secondary school (44% and 41% respectively). There was no significant difference in educational qualification between men and women. The level of employment was 43% for all participants while Nguni languages were mostly spoken as the home language (62%) and followed by Sotho languages (32%). The mean number of children was 2 (Table 1).

**Table 1:** Socio-demographic characteristics of study participants

	Men (*n* = 128)	Women (*n* = 302)	*P*-value	Total (*n* = 430)
Mean age (SD)	40.8 (8.7)	34.5 (7.8)	< 0.001	36.4 (8.6)
Age categories n (%)				
24 years and below	2 (1.6)	29 (9.6)	< 0.001	31 (7.2)
25–34 years	30 (23.4)	125 (41.4)		155 (36.1)
35–49 years	77 (60.2)	148 (49.0)		225 (52.3)
50 years and above	19 (14.8)	0 (0)		19 (4.4)
Marital status n (%)				
Single	42 (32.8)	148 (49.0)	0.004	190 (4.2)
Living together	35 (27.4)	81 (26.8)		116 (27.0)
Married	38 (29.7)	48 (15.9)		86 (20.0)
Divorced	7 (5.5)	9 (3.0)		16 (3.7)
Widowed	6 (4.7)	16 (5.3)		22 (5.1)
Education				
No schooling	17 (12.7)	26 (8.6)	0.375	43 (9.9)
Completed primary	62 (46.3)	128 (42.5)		190 (43.7)
Completed secondary	49 (36.6)	129 (42.9)		178 (40.9)
Tertiary	6 (4.5)	18 (6.0)		24 (5.5)
Currently Employed	65 (50.8)	120 (39.7)	0.034	185 (43.0)
Home Language				
Nguni (isiZulu, isiXhosa, isiNdebele, or siSwati)	80 (62.5)	186 (61.6)	0.930	266 (61.9)
Sotho (seSotho, Setswana, or isiPedi)	40 (31.3)	98 (32.5)		138 (32.1)
Others	8 (6.3)	18 (6.0)		26 (6.0)
Mean number of own children (SD)	2.3 (1.6)	1.9 (1.4)	0.0129	2.0 (1.4)

### HIV diagnosis, disclosure and treatment

The majority of participants (79.5%) knew about their HIV status for more than a year, with 9% finding out about their HIV status in the 6 months preceding the study. The duration since HIV diagnosis was not different between men and women (*p* = 0.969).

The majority of participants indicated that they had disclosed their HIV status (95%), with 97% of men and 94% of women indicating that they had disclosed their HIV status (*p* = 0.232). More women (71%) compared to men (54%) had disclosed their HIV status to family members; while more men (33%) compared to women (16%) disclosed their status to their partner (*p* < 0.001). Only 9% of study participants reported that they were living openly with HIV, with more men (12%) in this category.

Most participants (93%) were on antiretroviral treatment (HAART), although this differed significantly by gender (*p* = 0.018). Men (97.6%) were more likely to be on ART compared to women (91.3%) (*p* = 0.018). The majority of participants (77%) described their current health status as good or excellent. Only 4% perceived their health to be poor. Perceived differences in health status by gender were not statistically significant.

### Reproductive desire and contraception use

Among study participants, 46% (95% CI: 41.4–50.9) expressed a desire to have children in the future, 44% indicated that they do not wish to have children in the future, while 10% were unsure. There were no significant differences in reproductive desire by gender (*p*-value = 0.582), although a slightly higher proportion of men (50%) compared to women (44%) expressed the wish to have children in the future (Table 2).

**Table 2:** Participants’ expressed desire for children

	Men (*N* = 128)	Women (*N* = 302)	*P*-value	Total (*N* = 430)
Wish to have children in future n (%)				
No	52 (41.3)	137 (45.5)	0.582	189 (44.3)
Yes	63 (50.0)	134 (44.5)		197 (46.1)
Unsure	11 (8.7)	30 (10.0)		41 (9.6)

Although 189 of respondents (44%) indicated that they did not wish to have children in the future, only 71 reported the use of contraceptives.

### Reasons for wanting children

**Fig. 1:**
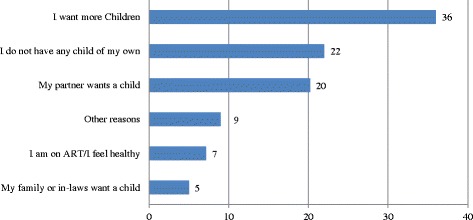
Reasons for wanting children

Figure 1 shows the participants’ reasons for wanting children. The commonest reasons were wanting more children (36%) and ‘I do not have any child of my own’ (22%).

### Knowledge of available reproductive methods

**Fig. 2:**
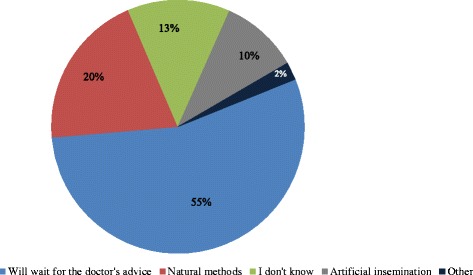
Knowlege of reproductive methods

The majority of the participants (55%) indicated that they will wait for the doctors’ advice on the appropriate fertility option and 20% of the respondents were in favour of the natural method of conception. Participants who expressed a desire for artificial insemination made up 10% while those who are not certain about the reproductive options available to them were 13% (Fig. 2).

### Factors influencing reproductive desire

In the multiple logistic regression analysis (Table 3), the main predictors of reproductive desire were: age below 49 years {35–49 years (AOR = 0.42; 95% CI: 0.24–0.72), below 25 years (AOR = 0.78; 95% CI: 0.23–2.59)}, being married (AOR = 2.21; 95% CI: 1.12–4.35) or living together (AOR = 2.78; 95% CI: 1.48–5.21) and having no child (AOR = 16.48; 95% CI: 5.94–45.74).

**Table 3:** Multiple logic regression model of factors associated with reproductive desire

Factor	Level	Unadjusted OR (95% CI)	Adjusted OR (95%CI)	*P*-value
Age	Less than 24 years	2.04 (0.72; 5.80)	0.78 (0.23; 2.59)	
	25–34 years	1 (baseline)	1 (baseline)	0.0001
	35–49 years	0.42 (0.24; 0.72)	0.42 (0.24; 0.72)	
	50 years and above	0.03 (0.004; 0.25)	0.04 (0.004; 0.33)	
Marital Status	Divorced / Widowed	0.42 (0.19; 0.94)	0.75 (0.27; 2.05)	
	Married	0.83 (0.49; 1.41)	2.21 (1.12; 4.35)	
	Living Together	1.86 (1.13; 3.07)	2.78 (1.48; 5.21)	
	Single	1	1	0.0022
No of children	None	15.57 (5.90; 41.10)	16.48 (5.94; 45.74)	
	One	5.65 (3.21; 9.92)	7.04 (3.69; 13.42)	
	2–3	1 (baseline)	1 (baseline)	<0.0001
	4 and above	0.26 (0.12; 0.58)	0.32 (0.14; 0.74)	
Highest Education Level	Less than primary	1 (baseline)	-	-
	Complete primary	2.5 (1.1; 5.5)		
	Completed secondary	3.7 (1.7; 8.3)		
	Tertiary	3.9 (1.3; 11.9)		
Health Status	Poor / Okay	1 (baseline)	-	-
	Good / Excellent	1.6 (1.0; 2.6)		

### Preferred setting for sessions on reproductive health information.

When asked about the preferred setting respondents would prefer to receive reproductive health information, majority chose seminar or workshop. There were no gender differences observed in the responses (*p* < 0.388). The preferred type of group make-up for sessions on reproductive health had majority (30%) preferring only men or women’s groups compared to individual (23%) session types.

## Discussion

The majority of the participants were 25–49 years of age, thus supporting existing evidence that HIV primarily affects the economically active section of the population [7, 39, 42, 43]. The findings underscore the importance of addressing the sexual and reproductive health needs of this sub-group of the population. There were high levels of unemployment and a high proportion of the population without private health insurance (uninsured) which implies dependence on the public health facilities [39, 40, 42, 43]. Hence, the importance of attending to the sexual and reproductive health needs of PLHIV, particularly in the public health sector.

In this study, 50% of men and 44% of women expressed a desire to have children. This expressed desire for children is similar to the findings of other studies. Cooper et al. found that in Cape Town, 55% of female and 43% of male participants in their study wanted children [19]. Similarly Beyeza-Kashesya et al. [17], found that 64% of female and 55% of male HIV positive participants expressed the desire for children. Rispel et al. found that the desire to have children was high amongst HIV-discordant couples interviewed in South Africa and Tanzania [22]. Chen et al. reported similar high rates in their study of fertility desires and intentions of PLHIV in the United States of America [24]. Oladapo et al. likewise reported a high expressed desire for children in their study in Nigeria [44].

Numerous studies have found that the desire for children among Africans is higher, compared to non-Africans [45–48]. Heard et al., reported that men and women of African ethnicity living in France expressed desire for children was three times higher compared to Europeans [27]. This was attributed to the socio-cultural background of people of African origin: for women, motherhood is considered as a high social status and identity, and as a prestige among peers while for men, on the other hand, fatherhood is critical as it ensures the continuation of the family name and lineage [47–53]. In this study, the population was homogeneous and ethnicity was not a considered variable.

In this study, the multi-variate analysis found that age below 49 years, being married or living together; and having no child were the predictors of reproductive desire (Table 3).

The study found that at age younger than 25 years of age, 80% of respondents expressed the desire for children, compared to 6% at age 50 years and above. These research findings are similar to those of other research studies [47–54]. Oladapo et al., found that the reason why younger HIV positive patients had a greater desire for children, was the socio-cultural pressure on young adults for fulfilment because of the fear of dying early from the disease [44].

Marriage or living together was also found to be one of the predictors of the desire for children. This may imply stability in a relationship and thus increase the desire for wanting children. However, the desire for children decreased among participants with more than one child compared to those with none, so these factors have to be looked at in combination. The inverse relationship between number of children and desire for children was reported by Cooper et al. and Chen et al. [19, 24].

A further argument could be advanced that those in marriage or living together might be of advanced age and/or having many children already and consequently may not desire more children. On the other hand, married couples might view the main outcome of marriage as having children and so will desire to have children despite their HIV status. The explanation for the increased desire for children among PLHIV who are either married or living together as independent variable observed in this study cannot be explained easily. Further research may be necessary to determine which of these variables are more strongly correlated to reproductive desires.

This study did not find self-reported health status or being on HAART to be a predictor of reproductive desire. This contrasts with other research findings [47, 55, 56]. In the study by Oladapo et al., ill-health due to advancing HIV infection, evidenced by low CD4 count, was found to be an independent predictor of fertility desire [44]. The socio-cultural fulfilment of child bearing made very sick individuals want to have children before dying [44].

In this study 44% of participants indicated that they do not want to have children. Among the reasons given by participants for not wanting children were: already had own children, lack of enough resources to care for another child, fear of a baby being born with HIV infection, old age and lack of a stable partner. However, among those not desiring children only 36% reported contraceptive use. This low usage of contraception was also reported by Smits et al. [44] and Nakayiwa et al. [47].

Most of the respondents who expressed a desire to have children were in favour of the natural method of reproduction (20%). This could be that the natural method is the only one known by the majority of participants. Although natural reproduction is recommended for PLHIV especially HIV sero-discordant couples on effective HAART regimen, there are specific requirements and guidelines which may not be understood by PLHIV [19, 57]. Hence, the research participants favouring natural methods may need guidance on the most appropriate method [19].

Thirteen present of respondents did not know the various types of reproductive options available. This lack of knowledge of PLHIV on fertility options may limit their ability to make informed reproductive decisions. It could be due to lack of information on reproductive issues among these PLHIV. The knowledge of available reproductive options by PLHIV has not been a focus of most research studies; hence, this finding could not be compared with the findings of other studies.

Most of the respondents (55%) wanted guidance from the medical personnel on the appropriate reproductive options available to them. This could be due to participants’ lack of knowledge about other options such as artificial insemination and assisted reproductive technologies (ART). In several instances the inadequacy on the part of HCWs on the provision of reproductive assistance and their negative attitudes towards PLHIV on sexual and reproductive health made it difficult to meet the needs of their patients [58, 59].

The possibility of adoption was not explored in our study but it would be interesting to understand how the situation in Ekurhuleni compares with the negative attitude towards adoption found in a study in Cape Town, South Africa [19].

This was a cross-sectional study carried out in a resource-limited district in Gauteng province, and the findings cannot be generalised to all districts in South Africa, except those with similar features to Ekurhuleni. The respondents in this research were those who attended the public clinics for care and treatment and therefore their responses may differ from those attending private facilities or private general medical practices. The study relied on self-reported information from PLHIV in a health care setting. Hence, there may be social desirability bias, as respondents may have been concerned about possible victimisation or negative consequences for subsequent care at the clinics. However, considerable efforts were made to use trained fieldworkers, and not the staff that provide care at these facilities where the study was conducted. All participants were given clear information and assured of their privacy and confidentiality during and after the interviews.

There are numerous strengths of this research study. This was one of the first studies to examine various aspects of sexual and reproductive health issues of HIV positive patients at a PHC level in Ekurhuleni district. A 100% response rate was obtained, and collection of data via face-to-face interviews ensured that the views of PLHIV who are not literate were elicited. This study also determined the preferred setting for information sharing and counselling on reproductive desires, an element hitherto unexplored in other studies on SRH and PLHIV.

This study found that there is expressed desire for children among PLHIV and their knowledge on the available reproductive options was limited. However, the participants expressed desire for information on reproductive options was also high. The respondents in this study indicated preference for men or women group discussions on reproductive health information.

There are encouraging developments within South Africa: the national strategic plan (NSP) for HIV/ AIDS and tuberculosis, 2012–2016, overseen by the South African National AIDS Council (SANAC), provides guidance on interventions and activities that will change the incidence and prevalence of HIV, STIs and TB [60]. However, the NSP falls short of guidelines on sexual and reproductive needs of PLHIV [60]. In the short term, sexual and reproductive health service guideline/protocols on reproductive options should be made available to HCWs. All health professionals (doctors and nurses, and other categories) should receive training on the reproductive needs and rights of PLHIV in other to improve their capacity and change their attitudes towards the SRH needs of HIV positive patients [53]. The clinics should also be equipped with educational and demonstration tools and audio-visual materials and equipment to educate PLHIV. In the medium term, the next version of the NSP should include specific goals, objectives, and outcome measures for the sexual and reproductive health needs of PLHIV. This will enable, provincial and district health plans to address the reproductive needs of PLHIV in line with SANAC strategic plan.

## Conclusion

The study generated locally, context specific information in a health district in Gauteng Province on the choices of HIV patients with regards to reproduction and the motivating factors for these choices and as well as their knowledge of fertility options. The study findings suggest that HCPs at primary health care level are critical to ensure that the sexual and reproductive health service needs of PLHIV are met.

## Additional file

Additional file 1: Questionnaire: reproductive choices among HIV positive patients in Ekurhuleni district, Gauteng province. (DOCX 18 kb)

## Abbreviations

AIDS: Acquired Immune deficiency syndrome
ART: Assisted reproductive technology
ARV: Antiretroviral
CHC: Community health centers
DHS: District health system
HCPs: Health care providers
HCWs: Health care workers
HIV: Human Immunodeficiency Virus
IVF: In-vitro fertilisation
NSP: National strategic plan
PHC: Primary health care
PLHIV: People living with HIV
SANAC: South African National AIDS Council
SRH: Sexual and reproductive health
STI: Sexually transmitted infection
TB: Tuberculosis
UNC: Universal health coverage
WHO: World Health Organisation
