# Supplementary material for: Factors influencing reproductive choices of HIV positive individuals attending primary health care facilities in a South African health district
Source: BMC Public Health. 2017 Jun 2;17:540. doi: 10.1186/s12889-017-4432-3 (PMC5457556; doi:10.1186/s12889-017-4432-3)
Supplement: Additional file 1: — Questionnaire: reproductive choices among HIV positive patients in Ekurhuleni district, Gauteng province. (DOCX 18 kb) [file 12889_2017_4432_MOESM1_ESM.docx]

**QUESTIONNAIRE: REPRODUCTIVE CHOICES AMONG HIV POSITIVE PATIENTS IN EKURHULENI DISTRICT, GAUTENG PROVINCE.**

**For official use only**

| 1. 2 | **Sub-district** |  | **O** ESDR 1  **O** SSDR 2  **O** NSDR 3 |
| --- | --- | --- | --- |
|  | **Health Facility ID** |  | \|  \|  \|  \| \| --- \| --- \| --- \| |
| 1. 3 | **Participant number** |  | \|  \|  \|  \|  \| \| --- \| --- \| --- \| --- \| |
| 1. 8 | Date of interview: | DD/MM/YY | \|  \|  \|  \|  \|  \|  \| \| --- \| --- \| --- \| --- \| --- \| --- \| |
| 1. 12 | **Was the interview completed?** | **O** No 0  **O** Yes 1 | \|  \| \| --- \| |
| 1. 15 | Date checked: | DD/MM/YY | \|  \|  \|  \|  \|  \|  \| \| --- \| --- \| --- \| --- \| --- \| --- \| |

SECTION 1: BACKGROUND CHARACTERISTICS

| **For official use only** |  |  |  |
| --- | --- | --- | --- |
| \|  \|  \| \| --- \| --- \| | **101** | How old are you**?** *(in completed years)* | ______ years |
| \|  \| \| --- \| | **102** | Gender | 🞎 Male…1  🞎 Female…2 |
| \|  \| \| --- \| | **103** | What is the main language that you speak at home? | **O** English…1  **O** isiZulu, isiXhosa, isiNdebele, or siSwati…2  **O** seSotho, Setswana, or sePedi…3  **O** Other…9 |
| \|  \| \| --- \| | **104** | What is your current marital status? | 🞎 Married...1  🞎 Living together...2  🞎 Single...3  🞎 Divorced...4  🞎 Widowed...5 |
| \|  \| \| --- \|  \|  \| \| --- \| | **105**  **106** | Do you have children of your own?  How many children do you have of your own? | 🞎 No... 0-go to 107  🞎 Yes...1  ______ |
| \|  \| \| --- \| | **107** | 1. What is your highest educational qualification? | 🞎 No schooling...1  🞎 Completed primary school (grade 7 or standard 5) ...2  🞎 Completed secondary school (grade 12 or matric)...3  🞎 Diploma....4  🞎 University degree ...5  🞎 Other....9  Specify:____________________ |
| \|  \| \| --- \| | **108** | Are you currently employed? | 🞎 No... 0 ---go to 201  🞎 Yes...1 |
| \|  \| \| --- \| | **109** | 1. What kind of work do you do? | Specify:____________________ |

*SECTION 2: HIV DISCLOSURE AND TREATMENT*

| **For official use only** |  |  |  |
| --- | --- | --- | --- |
| \|  \| \| --- \| | **201** | When were you diagnosed as HIV positive? | **O** less than 6 months ago--1  **O** 6-12 months ago--2  **O** more than one year ago--3 |
| \|  \| \| --- \| | **202** | Have you told anyone else about your HIV status? | **O** No 0---- go to 204  **O** Yes --1 |
| \|  \| \| --- \| | **203** | Who have you told about your HIV status | **O-**no one--0  **O** Partner only--1  **O** Own family member only(e.g. sister, brother, mother)--2  O Friends only--**3**  **O** Living openly with HIV--4  **O** Other--9  specify ----------------- |
| \|  \| \| --- \| | **204** | Are you currently on anti-retroviral treatment? | **O** No-- 0---go to 206  **O** Yes--1 |
| \|  \| \| --- \| | **205** | How long have you been on anti-retroviral therapy? | **………………….. months** |
| \|  \| \| --- \| | **206** | How would you rate your current health status | O Poor—1  O Satisfactory/Okay—2  O Good—3  O Excellent—4 |
| \|  \| \| --- \| | **207** | Have you experienced any form of discrimination as a result of your HIV status? | **O** No--0  **O** Yes--1 |

*SECTION 3: REPRODUCTIVE CHOICES*

| **For official use only** |  |  |  |
| --- | --- | --- | --- |
| \|  \| \| --- \| | **301** | Which best describes your sexual relationship at this time | O Not having sex--0 ---go to 303  O Having sex with one partner only--1  O Having sex with more than one partner--2 |
| \|  \| \| --- \| | **302** | In the past 12 months, of all times you had sex with your partner (s), how often did you use a condom? | O Never--0  O Sometimes--1  O Always--2  O Did not have a main partner--9 |
| \|  \| \| --- \| | **303** | Do you wish to have a child/children in future | **O** No--0  **O** Yes—1- go to 306  **O** Unsure/don’t know --3  **O Other--**9  Specify…………………… |
| \|  \| \| --- \| | **304** | If you do not wish to have a child/ children, what form of contraception are you using? | **O** Oral contraceptive--1  **O** Injection--2  **O** Other--9  Specify……………………………. |
| \|  \| \| --- \| | **305** | What are the reasons that you do not wish to have children? | O I already have children of my own--1  O My partner does not want a child--2  O I am worried that the baby will be HIV positive--3  O The doctor or sister advised me against it--4  O I do not have money to look after a baby--5  O I am too sick to look after a baby--6  O I am worried about stigma or discrimination--7  O I do not have a stable partner at present--8  O Other--9  Specify…………………….. |
| \|  \| \| --- \| | **306** | Could you tell us about the reasons that you want to have a child/ children? | O I do not have a child/ children of my own--1  O My partner wants a child--2  O My family or in-laws want a child--3  O I am worried that people will gossip about me if I do not have a child--4  O I feel healthy--5  O I am now on ART--6  O The sister or doctor spoke to me about having children--7  O Other--9  Specify……………………… |
|  | **307** | How do you plan to have a child? | O I will wait for the doctor to advise me/us--1  O Natural methods --2  O Artificial insemination--3  O I don’t know--4  O Other--9  Specify……………………… |

**Section 4: Clinic services**

| **For official use only** |  |  |  |
| --- | --- | --- | --- |
| \|  \| \| --- \| | 401 | In the past 12 months, have you talked to a doctor or nurse about having a baby? | **O** No--0 ---go to 404  **O** Yes--1 |
| \|  \| \| --- \| | 402 | In the past 12 months, has any doctor talked to you about having a baby? | **O** No--0  **O** Yes--1 |
| \|  \| \| --- \| | 403 | In the past 12 months, has any nursing sister talked to you about having a baby? | **O** No--0  **O** Yes--1 |
| \|  \| \| --- \| | 404 | Have you attended any information, session on any of the following: |  |
| \|  \| \| --- \| | a | *Family planning or contraception for People Living with HIV* | **O** No--0  **O** Yes--1 |
| \|  \| \| --- \| | b | *Counselling on how to lower the risk of transmitting HIV to others* | **O** No--0  **O** Yes--1 |
| \|  \| \| --- \| | c | *Prevention of mother-to-child transmission of HIV* | **O** No--0  **O** Yes--1 |
| \|  \| \| --- \| | d | *Different ways of having a baby when one is HIV positive (e.g. artificial insemination or sperm washing)* | **O** No--0  **O** Yes--1 |
| \|  \| \| --- \| | e | *ART and having a baby* | **O** No--0  **O** Yes--1 |
| \|  \| \| --- \| | f | *Other, specify----------* | **O** No--0  **O** Yes 1 |
| \|  \| \| --- \| | 405 | Would you be interested in attending a session on sexual relationships for PLHIV? | **O** No--0  **O** Yes--1 |
| \|  \| \| --- \| | 406 | Would you be interested in attending a session on family planning or contraceptives for PLHIV? | **O** No--0  **O** Yes--1 |
| \|  \| \| --- \| | 407 | Would you be interested in attending a session on how PLHIV can have a child/ children? | **O** No--0  **O** Yes--1 |
| \|  \| \| --- \| | 408 | What type of setting would you prefer for this information (*Choose only one answer?)* | **O** Individual one-on-one counselling--1  **O** A women or men’s group--2  **O** A seminar or workshop--3  **O** Other--9  **Please specify………………** |

**SECTION 5. What additional information or support or services, if any, would you like to have available to you at the clinic?**

…………………………………………………………………………………………………

**THANK YOU VERY MUCH FOR YOUR PARTICIPATION**
